# Mechanisms Underlying the Regulation of Mitochondrial Respiratory Chain Complexes by Nuclear Steroid Receptors

**DOI:** 10.3390/ijms21186683

**Published:** 2020-09-12

**Authors:** Ami Kobayashi, Kotaro Azuma, Kazuhiro Ikeda, Satoshi Inoue

**Affiliations:** 1 Department of Systems Aging Science and Medicine, Tokyo Metropolitan Institute of Gerontology, Itabashi-ku, Tokyo 173-0015, Japan; akobayashi-jik@umin.ac.jp (A.K.); azumak@tmig.or.jp (K.A.); 2 Department of Geriatric Medicine, Graduate School of Medicine, The University of Tokyo, Bunkyo-ku, Tokyo 113-0075, Japan; 3 Division of Gene Regulation and Signal Transduction, Research Center for Genomic Medicine, Saitama Medical University, Hidaka, Saitama 350-1241, Japan; ikeda@saitama-med.ac.jp

**Keywords:** mitochondria, respiratory chain complex, respiratory chain supercomplex, oxidative phosphorylation (OXPHOS), nuclear receptor, NR3 class nuclear receptor, nuclear steroid receptor

## Abstract

Mitochondrial respiratory chain complexes play important roles in energy production via oxidative phosphorylation (OXPHOS) to drive various biochemical processes in eukaryotic cells. These processes require coordination with other cell organelles, especially the nucleus. Factors encoded by both nuclear and mitochondrial DNA are involved in the formation of active respiratory chain complexes and ‘supercomplexes’, the higher-order structures comprising several respiratory chain complexes. Various nuclear hormone receptors are involved in the regulation of OXPHOS-related genes. In this article, we review the roles of nuclear steroid receptors (NR3 class nuclear receptors), including estrogen receptors (ERs), estrogen-related receptors (ERRs), glucocorticoid receptors (GRs), mineralocorticoid receptors (MRs), progesterone receptors (PRs), and androgen receptors (ARs), in the regulatory mechanisms of mitochondrial respiratory chain complex and supercomplex formation.

## 1. Introduction

**Figure 1:**
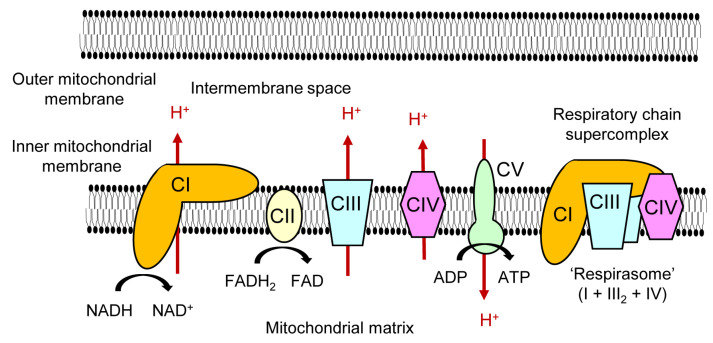
**Mitochondrial OXPHOS complexes and a respiratory chain supercomplex.** OXPHOS is carried out by five molecular complexes in the inner mitochondrial membrane, namely complex I (NADH ubiquinone oxidoreductase/NADH dehydrogenase; CI), complex II (succinate ubiquinone oxidoreductase/succinate dehydrogenase; CII), complex III (ubiquinol cytochrome *c* oxidoreductase/cytochrome *bc*_1_ complex; CIII), complex IV (cytochrome *c* oxidase; CIV), and complex V (ATP synthase; CV). Complexes I–IV are responsible for transferring electrons from NADH or FADH_2_ to molecular oxygen. In the process, protons (H^+^) translocate across the inner mitochondrial membrane from the mitochondrial matrix to the intermembrane space. The established proton gradient is essential for ATP generation by complex V. The movement of protons is indicated by red arrows. A certain portion of the mitochondrial respiratory chain complexes form a higher-order structure called a ‘supercomplex’. The supercomplex formed by one CI, two CIIIs, and one CIV is called ‘respirasome’.

In eukaryotic cells, mitochondria are involved in a wide variety of biological processes. One of the important roles of mitochondria is to supply energy to the cell via oxidative phosphorylation (OXPHOS). The mitochondrial OXPHOS consists of a series of five molecular complexes with enzymatic activities, namely, complex I (NADH ubiquinone oxidoreductase/NADH dehydrogenase), complex II (succinate ubiquinone oxidoreductase/succinate dehydrogenase), complex III (ubiquinol cytochrome *c* oxidoreductase/cytochrome *bc*1 complex), complex IV (cytochrome *c* oxidase), and complex V (ATP synthase). These OXPHOS complexes are composed of protein subunits that are encoded by either nuclear DNA or mitochondrial DNA (mtDNA) [1]. Genetic defects of OXPHOS-related genes that cause human diseases of mitochondrial energy metabolism are detected in every component of respiratory chain complexes [2], indicating that the function of each complex affects the output of whole OXPHOS process. It is also shown that the absence of complex III prevents the assembly of complex I and IV [3], which also indicates the cooperative regulation of OXPHOS complexes. In addition, some of the respiratory chain complexes form higher-order structures called ‘supercomplexes’ [4] (Figure 1). A supercomplex typically composed of one complex I, two complex IIIs, and one complex IV, is called ‘respirasome’. The biological significance of the mitochondrial respiratory chain supercomplex formation is efficient energy production [5,6,7], reduction in reactive oxygen species (ROS) generation [8,9], and/or stabilization of complex I [10].

The nuclear receptor superfamily consists of multiple transcription factors with a similar domain structure. Nuclear receptors were initially considered ligand-regulated transcription factors that mediate the actions of several hormones. With time, several transcription factors without known ligands, called ‘orphan’ receptors, were also characterized, and included in the nuclear receptor superfamily based on their structural properties. To date, 48 members of the nuclear receptor superfamily have been identified in humans [11].

In this review, we summarize the functional roles of nuclear steroid receptors (NR3 class nuclear receptors), including estrogen receptors (ERs), estrogen-related receptors (ERRs), glucocorticoid receptors (GRs), mineralocorticoid receptor (MR), progesterone receptors (PRs), and androgen receptor (AR), in the formation of mitochondrial OXPHOS complexes and respiratory chain supercomplexes in mammalian cells. Among them, ERs, PRs, and AR are the receptors for gonadal steroids, while GRs and MR are the receptors for corticosteroids. ERRs are orphan receptors whose physiological ligands do not exist or have not been identified. Gonadal steroids and corticosteroids are circulating hormones that affect multiple tissues or organs where nuclear steroid receptors are expressed.

From a phylogenetic point of view, nuclear receptors first originated in the metazoans [12]. This is supported by the fact that nuclear receptors are absent in fungi and plants. According to the endosymbiotic theory, mitochondria are the descendants of formerly free-living prokaryotes, which happened to live together within one cell in eukaryotes. Thus, the evolution of nuclear receptors, and their control of mitochondrial respiratory chains, highlights the cooperative interactions between the nucleus and mitochondria, which supply energy to the cell in a coordinated manner in response to the cellular environment.

## 2. Regulatory Mechanisms of Nuclear Receptors Affecting Mitochondrial Respiratory Chain Complexes

**Figure 2:**
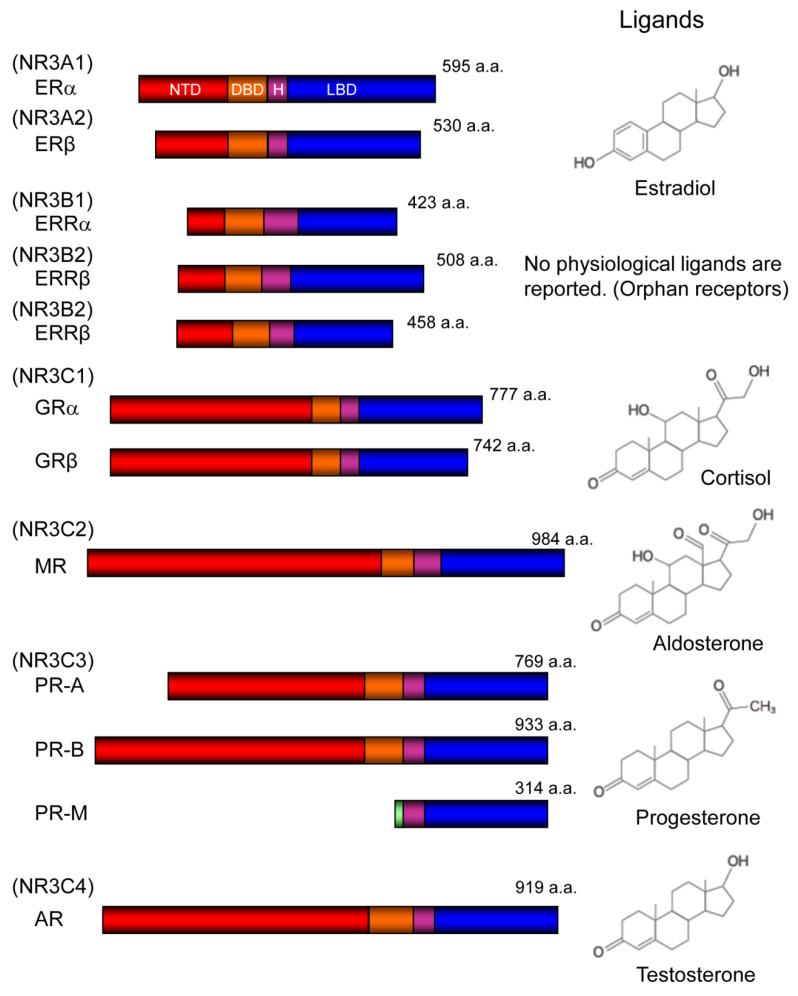
**NR3 class of nuclear receptors.** NR3 class of nuclear receptors and their corresponding ligands are shown. NR3 class of nuclear receptors are known as ‘nuclear steroid receptor’ because all the physiological ligands for them have a steroid backbone in their structural formula. Estrogen-related receptors (ERRs), for which physiological ligands do not exist or have not been identified, are referred to as orphan receptors. ER (estrogen receptor) subtypes (ERα and ERβ) and ERR subtypes (ERRα, ERRβ, and ERRγ) are coded in different genes. GR (glucocorticoid receptor) isoforms (GRα and GRβ) are splicing variants coded by the same gene. PR (progesterone receptor) isoforms (PR-A, PR-B, and PR-M) are derived from the same gene with distinctive transcription initiation sites. PR-M (mitochondrial progesterone receptor) contains a unique N-terminal amino acid sequence suggestive of a transmembrane domain (green). NTD; N-terminal domain (red), DBD; DNA-binding domain (orange), H; hinge region (purple), LBD; ligand-binding domain (blue), a.a.; amino acids.

Nuclear receptors share a common structure and are classified into six classes according to the phylogenetic tree based on sequence alignment [12]. In this review, we focus on the NR3 class of nuclear receptors, also known as ‘nuclear steroid receptors’ [13]. Except for estrogen-related receptors, all members of NR3 class nuclear receptors bind to steroid hormones as physiological ligands (Figure 2).

Nuclear receptors typically function as ligand-dependent transcription factors that regulate the expression of primary target genes. When the corresponding ligand binds to a nuclear receptor, the receptor binds to the regulatory DNA sequences (promotors or enhancers) of the primary target genes. In some cases, the interaction with the DNA sequences involves direct binding to short, distinctive sequences called hormone response elements (HREs). Alternatively, the interaction can be indirect tethering on other transcription factors. When nuclear receptors bind to the DNA sequence, they often form a homodimer (with the same nuclear receptor) or heterodimer (with another nuclear receptor). By binding regulatory DNA sequences, nuclear receptors interact with cofactors such as coactivators and corepressors, and subsequently affect the transcription of primary target genes. If the primary target gene happens to be another transcription factor, it affects the transcription of secondary target genes.

A different mode of nuclear receptor action is known, which is not mediated by transcriptional regulation. Nuclear receptors can interact with some signal transducing molecules in the cytosol, often in a ligand-dependent manner. Since the transcription of genomic DNA is not involved, this mode of action is called a nongenomic action [14], in contrast to the genomic action in which the nuclear receptor acts as a transcription factor. The characteristic feature of the nongenomic action is the rapid alteration of signal transducing molecules, which differs from the genomic action involving transcriptional and subsequent translational changes.

In terms of mitochondrial regulation, some nuclear receptors are reported to bind to mtDNA and regulate mitochondrial genes, as explained later. This mode of action can be regarded as a special type of ‘genomic’ action. Additionally, a special type of nongenomic action involves physical interactions of some nuclear receptors with mitochondrial proteins, primarily in the context of apoptosis regulation [15].

**Figure 3:**
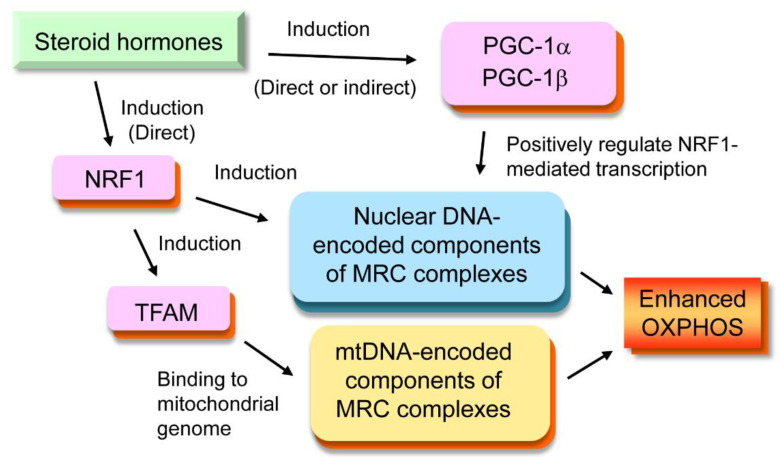
**Transcription factors regulating OXPHOS.** Nuclear respiratory factor 1 (NRF1), mitochondrial transcription factor A (TFAM), peroxisome proliferator-activated receptor γ coactivator -1α (PGC-1α), and peroxisome proliferator-activated receptor γ coactivator -1β (PGC-1β) are transcription factors which often mediate the effects of steroid hormones on OXPHOS. The *NRF1* gene possesses hormone response elements for some nuclear receptors in its promoter region and can be directly regulated by steroid hormones. TFAM is a secondary induced factor that exerts its effects on mtDNA. PGC-1α and PGC-1β are co-regulators that positively regulate NRF1-mediated transcription. MRC stands for mitochondrial respiratory chain.

In the genomic regulation of OXPHOS, several transcription factors are regulated in common by different nuclear receptors (Figure 3). One such transcription factor is the nuclear respiratory factor 1 (NRF1). Regulatory regions of the *NRF1* gene are reported to have HRE for some nuclear receptors, including estrogen response element (ERE) [16], ERR response element (ERRE) [17], and peroxisome proliferator response element (PPRE) [18]; therefore, this gene can be the primary target for these nuclear receptors. NRF1 stimulates the transcription of nuclear-encoded components of mitochondrial respiratory chain proteins. Another commonly regulated transcription factor is the mitochondrial transcription factor A (TFAM). *TFAM* is one of the genes up-regulated by NRF1 [19]; thus, this transcription factor can be a secondary target gene for some nuclear receptors, such as ERRs, GRs, PRs, and AR. TFAM translocates to the mitochondria and binds to the mitochondrial genome in a sequence-independent manner [20]. It has been reported that TFAM protects mtDNA, increases the amount of mtDNA, and induces the transcription of mtDNA-encoded OXPHOS proteins [21]. Moreover, peroxisome proliferator-activated receptor γ coactivator -1α (PGC-1α) and peroxisome proliferator-activated receptor γ coactivator -1β (PGC-1β) are other transcription factors commonly regulated by nuclear receptors. Peroxisome proliferator-activated receptor gamma (PPARγ) and ERRα are shown to induce transcription of PGC-1α directly by binding their response elements in the promoter region of PGC-1α [22,23]. Other members of nuclear steroid receptors, such as ERs, MR, and AR, also regulate expression of PGC-1α or PGC-1β directly or indirectly as described later. PGC-1α and PGC-1β activate transcriptional factors such as PPARγ, ERRα, and NRF1 by physical association with them [24,25]. Thus, PGC-1α and PGC-1β promote expression of OXPHOS-related proteins by positively regulating NRF1-mediated transcription. In addition, they also form a positive autoregulatory loop; their own transcription is induced by activation of PPARγ or ERRα.

## 3. ERs in the Regulation of Mitochondrial OXPHOS Complexes

Estrogen is a sex steroid hormone involved in a plethora of biological functions related to female reproductive tissues. It also affects several nonreproductive tissues in both sexes, where ERs are expressed. In addition, estrogen is a promoting factor for ER-positive breast cancer. Two subtypes of estrogen receptors are known, namely, estrogen receptor alpha (ERα; NR3A1) and estrogen receptor beta (ERβ; NR3A2), which are coded by different genes. Both of them belong to the nuclear receptor superfamily.

Estrogen affects the expression of genes involved in mitochondrial respiratory chain complexes and OXPHOS [26]. These effects are mainly mediated by the genomic action of ERs in the nucleus. A previous study showed that NRF1 is a direct target of ERs, and a functional estrogen response element (ERE) exists in the *NRF1* promoter region, whereby both ERα and ERβ can bind [16]. In this study, NRF1 protein was induced by estrogen stimulation in MCF-7 human breast cancer cells and H1797 human lung cancer cells. As a consequence, *TFAM* and two mtDNA-encoded genes, cytochrome *c* oxidase subunit I (*Cox1*) and NADH dehydrogenase subunit I (*NDI*), were induced by estrogen [14]. Additionally, estrogen has been shown to induce PGC-1β in rat brain, mouse liver, and human hepatocellular carcinoma HepG2 cells [27,28]. This is accompanied by increased protein levels of mitochondrial OXPHOS complexes I, III, and V in the rat brain, and up-regulation of *Cox1* expression with enhanced ATP production in HepG2 cells. Another example of the primary ER target affecting mitochondrial respiratory chain is COX7RP (cytochrome *c* oxidase subunit 7a-related polypeptide, also known as COX7A2L/SCAF1). *COX7RP*, which possesses a perfect palindromic ERE in the intron 1, was identified as an estrogen responsive gene in MCF-7 cells by genomic-binding site cloning [29]. Later, it was found to function as a mitochondrial respiratory chain supercomplex assembly-promoting factor in murine skeletal muscles [5] as well as in MCF-7 cells [30]. In the latter study, estrogen was shown to induce cytochrome *c* oxidase (COX) activity and mitochondrial ATP content, which was attenuated by knocking down of COX7RP [30].

Notably, nuclear receptors do not always up-regulate their target genes; suppressive modes of regulation also exist. One of the examples of down-regulated ERα target genes related to OXPHOS is uncoupling protein 3 (*Ucp3*). UCP3 is a member of uncoupling proteins which localize to the mitochondrial inner membrane and uncouple OXPHOS via proton leakage, leading to energy dissipation. It was observed that ovariectomy in female mice increased the expression of *Ucp3*, which was suppressed by estrogen treatment [31]. Overexpression of constitutively active ERα (caERα) suppressed Ucp3 expression, whereas treatment with ICI182,780, an ER antagonist, induced *Ucp3* expression in C2C12 myoblastic cells, indicating that this regulation was mediated by ERα. Alternatively, the effect of ER-mediated transcriptional regulation of OXPHOS can be explained by the induction of another nuclear receptor, NR4A1 [32]. Overexpression of caERα up-regulated NR4A1 expression and ATP content in C2C12 cells. It was shown that knockdown of NR4A1 in pancreatic β-cells resulted in a significant decrease in mitochondrial respiration, accompanied with decreased expression and protein levels of SDHB, a subunit of mitochondrial respiratory chain complex II [33]. It could be inferred that OXPHOS’ promoting effect of NR4A1 in skeletal muscle is also mediated by SDHB induction.

Direct transcriptional regulation of mtDNA-encoded genes by ERs is also proposed. This possibility is based on the detection of ERs in mitochondria [34]. The existence of ERs in mitochondria was reported by multiple methods, including mass spectrometric analysis of human heart mitochondria [35], fluorescence microscopic analysis of human tumor cells [36], immunoprecipitation of mtDNA and western blotting using MCF-7 cells [37], and electron microscopic analysis using human fetal brown adipose tissue (BAT) [38]. However, it remains to be elucidated whether they are functional ERs.

The nongenomic actions of ERs have been implicated in many physiological and pathological processes [14]. However, information on the direct link between the nongenomic action of ERs and OXPHOS is limited. In human endometrial cells, estrogen induced rapid phosphorylation of p38 MAPK (mitogen-activated protein kinase) could be suppressed by ICI182,780 [39], indicating that ER mediates this reaction. On the contrary, macrophages of MAPK phosphatase-1 (MKP-1, also known as DUSP1) deficient mice exhibited higher expression of NRF1, TFAM, and PGC-1α [40]. Considering that MKP-1 preferentially dephosphorylates p38 MAPK [41], there may be a link between OXPHOS and MAPK signaling regulated by nongenomic action of ER.

**Figure 4:**
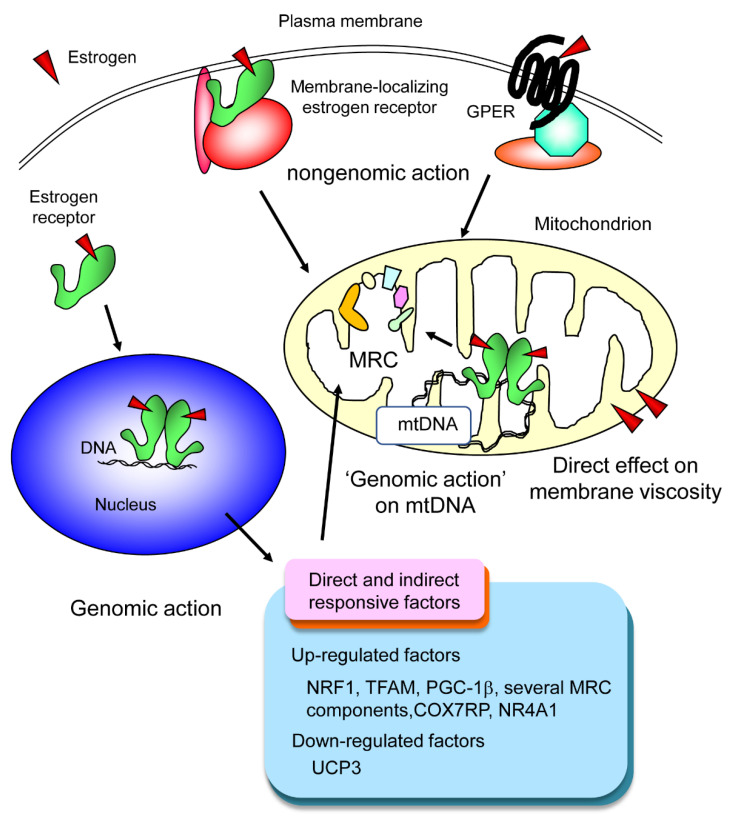
**Regulation of OXPHOS by estrogen.** Estrogen can regulate OXPHOS in several different manners. First, estrogen receptor (ER)-mediated classical genomic action regulates several nuclear DNA-encoded OXPHOS-related genes. Second, estrogen receptors found on mtDNA may regulate mtDNA-encoded OXPHOS-related genes, which can also be regarded as a kind of genomic action. Third, estrogen can affect OXPHOS through nongenomic action. This mode of action is mediated either by a small portion of ER (as a nuclear receptor) localizing at the plasma membrane or by a different membrane receptor called GPER (G-protein coupled estrogen receptor), also known as GPR30. Lastly, a receptor-independent mechanism is reported. In this mechanism, estrogen incorporated in the mitochondrial membrane alters its microviscosity, eventually affecting the activities of mitochondrial respiratory chain (MRC) complexes.

The significance of ER-mediated estrogen signaling in OXPHOS has been demonstrated in various studies involving knocking out or knocking down of ERs. For example, CD4+ T cell-specific knockout of ERα led to impaired OXPHOS [42]. Muscle-specific ERα knockout mice displayed impaired ATP production [43]. Furthermore, knocking down of ERβ in endometrial cells resulted in decreased expression of NRF1, TFAM, mtDNA-encoded COX1, and mtDNA-encoded ATP6 [44]. However, not all effects of estrogen on OXPHOS seem to be mediated via ERs. The involvement of G-protein coupled estrogen receptor (GPER, also known as GPR30) [45] and the direct effect of estrogen molecules on mitochondrial membrane viscosity [46] have been reported as ER-independent mechanisms of estrogen actions on mitochondrial respiratory chain. Thus, the regulation of OXPHOS by estrogen is potentially mediated by various pathways (Figure 4).

The effects of estrogen on OXPHOS explained above may have implications in human diseases such as neurodegenerative disorders, sarcopenia, and breast cancer. By the proteomic analysis of the white matter of elderly people with Alzheimer’s disease with cerebrovascular disease, sexual dimorphism of mitochondrial proteome was observed, where several OXPHOS-related proteins were down-regulated in postmenopausal women [47]. This may explain the beneficial effect of estrogen replacement therapy in several observational studies [48]. Estrogen replacement is suggested to have a beneficial effect also on sarcopenia. According to the meta-analysis of estrogen-based hormonal therapy for post-menopausal women, estrogen affected beneficially on muscle strength [49]. In the animal experiment, estrogen treatment was shown to recover exercise endurance impaired by ovariectomy [31]. Improved OXPHOS by estrogen explained above may be one of the mechanisms underlying the beneficial effect of estrogen on muscular tissue. In relation with breast cancer, shorter disease-free survival was observed in the patients of breast cancer with higher expression of COX7RP [30], suggesting that induction of COX7RP and increased OXPHOS by estrogen in the breast cancer tissue may partly explain the tumor promoting function of estrogen.

## 4. ERRs in the Regulation of Mitochondrial OXPHOS Complexes

ERRs comprise the NR3B subfamily, consisting of ERRα (NR3B1), ERRβ (NR3B2), and ERRγ (NR3B3), which are coded in different genes. ERRs were discovered in a screen designed to identify novel steroid hormone receptors related to ERα; hence, they were called ERR. Until now, they are recognized as orphan nuclear receptors since no natural ligands have been discovered. Among them, ERRβ and ERRγ are known to have synthetic ligands. GSK4716 and GSK9089 act as agonists for ERRβ and ERRγ [50], while diethylstilbestrol and 4-hydroxytamoxifen are reported to function as inverse agonists for ERRγ [51]. ERRs are transcriptionally active even without an agonistic ligand [52], and their transcriptional regulation is dependent on coregulators such as PGC-1α [53], mammalian target of rapamycin (mTOR) [54], growth arrest and DNA-damage-inducible protein 45γ (GADD45γ) [55], prospero-related homeobox 1 (PROX1) [56], and nuclear receptor corepressor 1 (NCoR1) [57].

Chromatin immunoprecipitation (ChIP)-sequencing analyses with gene expression analyses in the mouse skeletal muscle and liver revealed that ERRα up-regulated multiple OXPHOS-related genes in cooperation with PGC-1α or mTOR [53,54]. Interestingly, ERRα occupied regulatory regions of more than 70 kinds of OXPHOS-related genes [54], suggesting its significant influence on OXPHOS.

Genome-wide analyses of ERRα and ERRγ revealed their direct and overlapping binding in promoter regions of a large number of PGC-1α targets, including nuclear DNA-encoded OXPHOS-related genes [58]. In a study involving ERRα single knockout mice, ERRγ single knockout mice, and ERRα-ERRγ double knockout mice, expression of DNA-encoded OXPHOS-related genes were significantly down-regulated in the heart muscle of ERRα-ERRγ double knockout mice [59], indicating the compensatory mechanism of these two ERRs and their importance in the regulation of OXPHOS. This compensatory mechanism was also demonstrated using adipose tissue-specific ERRα and ERRγ knockout mice. In BAT derived from mice lacking both ERRα and ERRγ, expression of representative components of mitochondrial OXPHOS complexes I, II, III, and IV decreased dramatically, while modest but significant reductions were observed in components of complexes I, IV, and V from mice lacking only ERRα [60]. Similar results showing the compensatory function of ERRs were obtained using BAT lacking all subtypes of ERRs, namely, ERRα, ERRβ, and ERRγ [61]. In that report, reduced expression of ERRβ in BAT was observed, making it difficult to assess the contribution of ERRβ in OXPHOS regulation.

## 5. GRs in the Regulation of Mitochondrial OXPHOS Complexes

Glucocorticoid is one of the steroids secreted by adrenal glands in response to several types of stress. Low-dose glucocorticoid treatment causes a short-term increase in mitochondrial oxidation [62], which reflects increased energy demand during an acute stress response. Conversely, high-dose treatment or chronic treatment has a suppressive effect on mitochondrial oxidation [63]. The major molecules of GRs are GRα and its splicing variant, GRβ; both of them belong to nuclear receptor superfamily and are collectively classified as NR3C1. GRβ has a dominant-negative effect due to the truncated LBD [64]. Since effects of glucocorticoid on OXPHOS are observed in BAT cells, leukocytes, and neuronal cells, it is suggested that GRs are involved in the stress response within these cells.

One of the suggested mechanisms through which glucocorticoids affect OXPHOS is the genomic regulation of GR-targeted nuclear-encoded genes. It was shown that expression levels of nuclear-encoded genes including *Nrf1*, *Tfam*, and genes coding a few components of respiratory chain complex IV were elevated in primary BAT cells derived from mice lacking 11β-HSD1, which converts inactive 11-dehydrocorticosterone to active corticosterone in rodents (and cortisone to cortisol in human), compared with the expression in BAT cells from wild-type mice [64]. In another study involving BAT-specific GR knockout mice, expression of *Tfam* was elevated in BAT derived from BAT-specific GR knockout mice, as demonstrated by microarray analysis; however, expression of *Ppargc1a* (coding PGC-1α) and *Nrf1* remained unchanged [65]. These results reflect the suppression of BAT function, such as thermogenesis, by chronic glucocorticoid exposure. In contrast, microarray analysis following low-dose corticosterone treatment of primary rat cardiomyocytes for 24 h revealed that OXPHOS-related genes were not regulated by the treatment [66]. This may indicate that the genomic action of GR on nuclear-encoded OXPHOS-related genes is dependent on the cell type or species. Another example of GR-mediated genomic action on nuclear-encoded OXPHOS genes is the induction of glucocorticoid-induced leucine zipper (GILZ). GILZ is a glucocorticoid-induced transcription factor expressed in T-lymphocytes and is encoded in nuclear DNA with glucocorticoid response elements (GREs) in its promoter region [67]. In mouse leukemia cells, overexpression of GILZ caused enhanced oxygen consumption and higher cellular ATP level [68], indicating the OXPHOS-promoting effect of this protein.

Evidence from multiple studies suggests that GR affects OXPHOS via ‘genomic’ action on mtDNA. It was pointed out that human and rat mtDNAs contain multiple sequences similar to that of the nuclear glucocorticoid response element (GRE) [63]. In the rat hippocampus, acute immobilization stress led to decreased expression of several mtDNA-encoded genes, which was not observed in adrenalectomized rats [69]. This study reported GR binding to the D-loop region of mtDNA, a noncoding regulatory region on mtDNA, by a chromatin immunoprecipitation (ChIP) experiment [69].

Little is known about the nongenomic effects of glucocorticoid on OXPHOS. One report suggested that OXPHOS is controlled by G-protein-coupled receptor (GPCR) rather than membrane localized classical GR (which can also mediate genomic action in the nucleus). In HepG2, 10 min dexamethasone treatment affects the enzymatic activities of mitochondrial respiratory chain complexes I, II, and III [70]. This rapid effect could be blocked by GDPbS, an antagonist of G_a_ protein, suggesting GPCR dependence. Moreover, 8 h treatment of dexamethasone coupled with bovine serum albumin (dexa-BSA) significantly increased the respiration rates in HepG2 cells, which is in line with the involvement of either GPCR or membrane-localizing GR.

## 6. MR in the Regulation of Mitochondrial OXPHOS Complexes

Aldosterone is a steroid hormone that plays a central role in the maintenance of sodium homeostasis in kidneys. Life-threatening salt loss is caused by compromised action of aldosterone in human and by knockout of its receptor MR (NR3C2/Nr3c2) in mice [71]. Besides the regulation of sodium absorption in kidneys, aldosterone appears to play a vital role in the pathogenesis of heart failure. In several clinical studies, mineralocorticoid antagonists are shown to improve survival among patients with chronic heart failure and heart failure after myocardial infarction [72,73,74].

MR forms homodimer or heterodimer with GR and binds to DNA with HRE almost identical to GRE [75]. However, ChIP-seq experiments revealed that the majority of MR-binding sites on DNA do not possess typical GRE-like HRE [76,77]. Nongenomic actions mediated by MR are also reported [78].

The direct effects of MR signaling on OXPHOS are yet to be elucidated. However, several studies on aldosterone functions in the heart suggested implication of MR in OXPHOS regulation. For instance, the expression of MR was increased, while that of PGC-1α was decreased in the aging rat heart. Furthermore, in an aged heart muscle cell model using H9C2 rat cardiomyocytes treated with H_2_O_2_, pretreatment of eplerenone, an antagonist for MR, attenuated the decreased PGC-1α expression [79], which suggests the negative effect of aldosterone on OXPHOS during aging. In a study involving sheep model of atrial fibrillation induced by a pacemaker, protein expression of several components of mitochondrial respiratory chain I, II, III, and IV in the left atrial appendage was decreased, as shown by mass spectrometric analyses. Pretreatment with eplerenone attenuated the decreased expression of OXPHOS-related proteins caused by atrial fibrillation pacing [80]. In another study, the effect of aldosterone on human cardiac fibroblasts was evaluated. Aldosterone treatment of human cardiac fibroblasts decreased the expression of A-kinase anchor protein 12 (AKAP-12) [81], which was formerly identified by proteomic analysis as a protein negatively regulated by aldosterone [82]. Aldosterone treatment also suppresses the expression of PGC-1α. Overexpression of AKAP-12 increased PGC-1α expression in aldosterone-treated human cardiac fibroblasts [81], indicating the role of AKAP-12 in aldosterone function. In that report, prohibitin (PHB) was identified as another target of AKAP-12 mediated suppression by aldosterone. Since PHB is known as one of the mitochondrial respiratory chain supercomplex assembly-promoting factors [83], these results suggest that MR signaling affects respiratory chain supercomplex formation. 

## 7. PRs in the Regulation of Mitochondrial OXPHOS Complexes

Progesterone is a steroid hormone involved in the regulation of female reproductive processes. Elevated circulatory levels of progesterone are detected in the luteal phase of the menstruation cycle and during pregnancy. Two isoforms of nuclear receptors have been identified for progesterone, namely PR-A and PR-B, which are coded by the same gene and collectively classified as NR3C3. Unlike the longer PR-B form, PR-A lacks a 164 amino acid sequence in the N-terminus region as a result of distinctive transcription initiation; however, transcription of both isoforms is induced by estrogen [84]. PR-A and PR-B form homodimers or heterodimers [85], and PR-A exerts dominant negative effects on PR-B mediated transcription [86]. ChIP-seq analyses of human endometrium [87] and mouse uterus and ovary [88] revealed that PRs exert genomic action by binding progesterone response elements (PREs) or other transcription factor binding motifs. Nongenomic effects of PRs are also reported, which are mediated by the interaction of a proline-rich sequence motif within PRs and SRC homology 3 (SH3) domain in Src family tyrosine kinases leading to the activation of Src family tyrosine kinases [89].

Relatively high body temperature during the luteal phase is associated with the effects of progesterone on BAT mitochondria. Indeed, progesterone up-regulates the expression of norepinephrine induced uncoupling protein 1 (UCP1) [90], which is responsible for thermogenesis in BAT. It has been reported that *Tfam,* one of the nuclear-coded genes, is up-regulated in BAT by progesterone [91]. These results indicate the role of genomic action of progesterone in OXPHOS regulation.

Regarding the effects of progesterone in mitochondria, one isoform of PR called mitochondrial progesterone receptor (PR-M) is reported to localize in the outer mitochondrial membrane. PR-M lacks NTD and DBD of PR-A and PR-B. Instead, this isoform comprises a unique 16 amino acid sequence at N-terminus, followed by C-terminal side of other PRs, including hinge region and LBD [92] (Figure 2). The N-terminal amino acid sequence unique to PR-M mainly consists of hydrophobic amino acids, which is suggestive of a transmembrane domain, similar to the N-terminal sequence of other mitochondrial outer membrane proteins. Mitochondrial localization is shown by multiple methods, including fluorescence microscopic analysis, Western blot analysis, and electron microscopic analysis using human heart tissue or Cos-1 monkey kidney cells [93]. In MCF-10A breast epithelial cells where PR-M is expressed, but other PRs are not expressed, progesterone increased mitochondrial membrane potential and ATP production [94], indicating that ligand-dependent action of PR-M may exist. The biological significance of PR-M is pursued in several cell types, including a recent report on oocytes and embryo [95].

## 8. AR in the Regulation of Mitochondrial OXPHOS Complexes

Androgen is another sex steroid hormone playing vital roles in male reproductive tissues. In addition, androgen is a promoting factor for prostate cancer. It is also shown that androgen affects mitochondrial functions in tissues where AR (NR3C4), a member of nuclear receptor superfamily, is expressed.

There are several reports on the effect of testosterone (a typical androgen) deficiency or testosterone supplementation on the mitochondrial OXPHOS. For example, castration decreased ATP production in a rat model of myocardial infarction, which was ameliorated by testosterone replacement [96]. In male rat brains, castration resulted in reduced expression of NRF1, TFAM, PGC-1α, and components of respiratory chain complexes I, III, and IV [97]. Besides, testosterone replacement in castrated rats or aged male rats increased the expression of mtDNA-encoded component of complex I and plural subunits of complex V [98]. However, these studies do not precisely reflect functions of AR, because testosterone can be converted to estrogen by aromatase. Therefore, the results of testosterone replacement potentially include effects mediated by ERs unless aromatase inhibitor is used at the same time.

The importance of AR in OXPHOS regulation was demonstrated in a study utilizing AR knockout or mutant cells. In a study, induced pluripotent stem cells (iPSCs) were established from fibroblasts of a patient suffering from spinal and bulbar muscular atrophy (SBMA), which is caused by the expansion of a polyglutamine-coding CAG repeat in the first exon of AR gene. AR knockout iPSCs were also established. With chromatin immunoprecipitation (ChIP) assay using iPSCs derived motor neuron-like cells, dysregulated histone acetylation accompanied by reduced mitochondrial ATP production was observed in SBMA patient-derived cells and AR knockout cells [99]. This result suggested that genomic action of AR with proper epigenetic modifications has a promotive role for OXPHOS in motor neuron cells. The role of AR in female fertility is also emphasized. A previous study reported reduced fertility in granulosa cell (in ovary)-specific AR knockout mice [100]. Granulosa cells from systemic AR knockout mice were observed to have reduced ATP content compared to those from control mice [101]. Intriguingly, AR is shown to have suppressive effects on OXPHOS in prostate cancer cells. In AR knockout LNCaP cells (human prostate cancer cells), expression of TFAM and several nuclear DNA-encoded or mtDNA-encoded subunits of respiratory chain complexes were increased. In addition, mitochondrial respiratory chain supercomplex formation was also increased in AR knockout LNCaP cells [102].

## 9. Conclusions

In the present review, we described multiple nuclear receptors involved in mitochondrial respiratory chain complexes and supercomplexes through several pathways. Clarifying the mechanisms of nuclear receptors regulating mitochondrial respiratory chain complexes and supercomplexes will help identify therapeutic targets for various diseases, such as heart failure and sarcopenia, where OXPHOS is deeply involved. Discoveries of direct pathways of hormones and nuclear receptors, or identification of entirely new nuclear receptors affecting complex and supercomplex assembly factors and related molecules, are expected in future studies. Some nuclear receptors mentioned in the present review can be the targets for innovative treatment or prophylactic agents for OXPHOS-related conditions. Despite various clinical trials and moderate therapeutic use of agonists and antagonists of nuclear receptors, most of them have not yet been introduced in clinical practice. Further research on nuclear receptors and regulating pathways toward mitochondrial respiratory chain complexes and supercomplexes would be required for the discovery of novel therapeutic approaches for OXPHOS-related diseases.
